# Microscopic Electron Dynamics in Metal Nanoparticles for Photovoltaic Systems

**DOI:** 10.3390/ma11071077

**Published:** 2018-06-25

**Authors:** Katarzyna Kluczyk, Lucjan Jacak, Witold Jacak, Christin David

**Affiliations:** 1 Department of Quantum Technologies, Faculty of Fundamental Problems of Technology, Wrocław University of Science and Technology, 50-370 Wrocław, Poland; katarzyna.kluczyk@pwr.edu.pl (K.K.); lucjan.jacak@pwr.wroc.pl (L.J.); witold.aleksander.jacak@pwr.edu.pl (W.J.); 2 Madrid Institute for Advanced Studies in Nanoscience (IMDEA Nanoscience), C/Faraday 9, 28049 Madrid, Spain

**Keywords:** nanoparticles, microscopic electron dynamics, nonlocality, light interaction, theory and simulation

## Abstract

Nanoparticles—regularly patterned or randomly dispersed—are a key ingredient for emerging technologies in photonics. Of particular interest are scattering and field enhancement effects of metal nanoparticles for energy harvesting and converting systems. An often neglected aspect in the modeling of nanoparticles are light interaction effects at the ultimate nanoscale beyond classical electrodynamics. Those arise from microscopic electron dynamics in confined systems, the accelerated motion in the plasmon oscillation and the quantum nature of the free electron gas in metals, such as Coulomb repulsion and electron diffusion. We give a detailed account on free electron phenomena in metal nanoparticles and discuss analytic expressions stemming from microscopic (Random Phase Approximation—RPA) and semi-classical (hydrodynamic) theories. These can be incorporated into standard computational schemes to produce more reliable results on the optical properties of metal nanoparticles. We combine these solutions into a single framework and study systematically their joint impact on isolated Au, Ag, and Al nanoparticles as well as dimer structures. The spectral position of the plasmon resonance and its broadening as well as local field enhancement show an intriguing dependence on the particle size due to the relevance of additional damping channels.

## 1. Introduction

An accurate description of microscopic properties of metal nanoparticles (metal NPs—MNPs) is important to predict the optical response of e.g., molecules in close proximity to metal surfaces and resulting field enhancement and quenching effects. Nanoparticles as part of functionalized layers in sensing, spectroscopy [1] and light harvesting applications, photovoltaics [2,3,4,5,6,7] and photocatalysis [8,9,10,11,12], can improve the performance of such devices. They are efficient subwavelength scatterers improving the light trapping effect and MNPs provide, in particular, large local fields enhancing charge carrier generation, absorption, and light-induced effects from other nanostructures such as spectral conversion [13] or photoluminescence [14].

For over a hundred years, modeling of the optical properties of MNPs relies on classical electrodynamics. In highly symmetric cases (spherical and cylindrical NPs) analytic solutions are obtained within Mie scattering theory [15] using corresponding basis functions. The electric part E of the electromagnetic field creates a polarization field $P=\alpha (\epsilon_{0},\epsilon )E$ in solid matter, expressed in terms of the permittivities $\epsilon_{0}\left(\omega \right)$ and ϵ(ω) of the environment and the bulk material, respectively. This polarizability α , depending only on the optical response at a frequency ω , neglects microscopic electron interaction effects at the ultimate nanoscale arising not only from the quantum nature of the free electron gas in metals, but also from accelerated motion in the plasmon oscillation.

**Figure 1:**
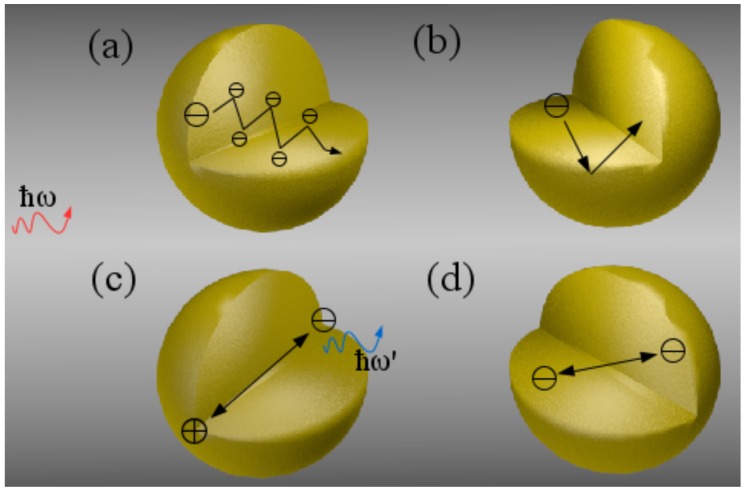
Illustration of sources of plasmon damping and electron interaction phenomena. (**a**) Electron-electron collisions in the bulk material; (**b**) Electron-surface collisions due to confinement; (**c**) Electron irradiation due to acceleration during plasmon oscillation; (**d**) Short-ranged electron-electron interactions, such as Coulomb force and electron diffusion.

Light-matter interaction involves processes within the electron subsystem in solids, crystals and molecules. Inhomogeneities on the length scale of the de Broglie wavelength $\lambda_{e}=\frac{h}{\sqrt{2mE}}$ produce scattering and interference effects of electrons which mutually interact with incoming light, see Figure 1a. Hereby, *h* is Planck’s constant, *m* is the (effective) electron mass which depends on the bulk material, and *E* is the energy of the electron wave. Typically, this wavelength is about 7.5 nm in solids at room temperature T=300 K, where $E=k_{B}T$ with the Boltzmann constant $k_{B}$ . For MNPs, the main source of electron scattering is the particle surface, see Figure 1b, where the surface-to-volume ratio indicates the relevance of such scattering events.

Microscopic interaction effects of electrons in metals are accurately described using first-principle methods, e.g., Density Functional Theory (DFT) [16,17,18]. These solve Schrödinger’s equation for a large, but finite number of electron wave functions from all atoms in the considered system. Unfortunately, even with strong approximations such as the Time Dependent Local Density Approximation (TDLDA), time-consuming algorithms limit their applicability to particles of a few nanometers in size [19,20,21]. Moreover, advances in fabrication of nanostructures along with experimental access to particle sizes and interparticle spacings below 10 nm led to the possibility of direct or indirect observation of such effects [22,23,24,25,26,27,28,29]. The situation described above resulted in increased interest in semi-classical approaches towards the incorporation of damping and interaction effects stemming from the quantum nature of charge carriers, illustrated in Figure 1. In this article, we present two such semi-classical approaches, the Random Phase Approximation (RPA) and Generalized Nonlocal Optical Response (GNOR), and ultimately combine them into a single framework to study their joint impact on MNPs of different materials, sizes and in different environments.

The original formulation of light scattering by a sphere by Gustav Mie [15] excludes microscopic dynamics of the conduction band electrons in bulk and surface effects. However, efforts to extend have been made since the 1970s [30,31,32,33,34,35,36,37,38,39]. Advanced semi-classical material models can be derived from perturbative theories [40,41], by separating the free electron dynamics from the core electron polarization via the hydrodynamic equation for an electron plasma [41,42,43,44,45,46,47,48,49,50,51,52,53,54,55,56,57,58,59], and from microscopic theories [60,61,62,63,64]. It should be noted that a major advantage of ab initio methods lies in their capability to account for the electron spill-out (evanescent tail of the electron wave functions) of the electron density into the surrounding dielectric medium. It was shown within the hydrodynamic framework that the electron spill-out can be adequately incorporated [57,65] and a current-dependent potential can be accounted for [66], which is, however, out of scope of the present study.

**Figure 2:**
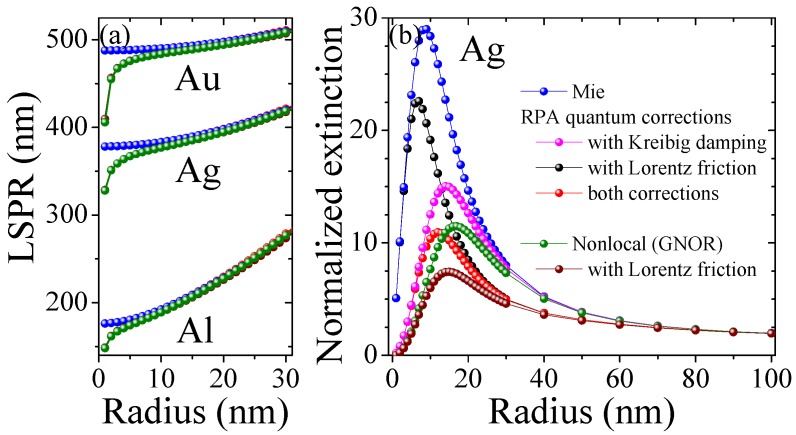
Impact of quantum corrections on single nanoparticles. (**a**) Spectral position of the localized surface plasmon resonance (LSPR) for gold, silver and aluminum; (**b**) Extinction cross section normalized to the surface of a hemisphere for silver evaluated at the respective LSPR wavelengths from (**a**).

**Figure 3:**
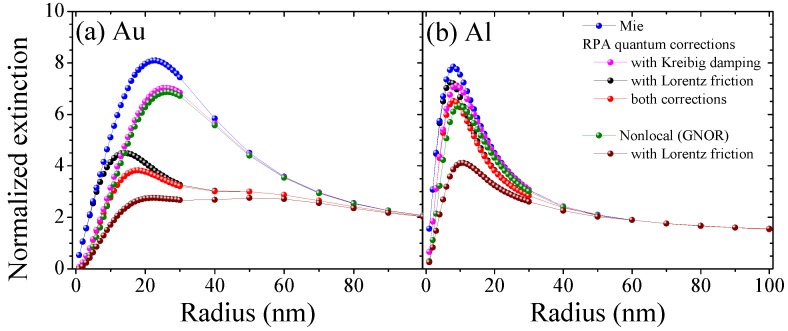
Extinction cross section normalized to the surface of a hemisphere for isolated (**a**) gold and (**b**) aluminum nanoparticles evaluated at the respective LSPR wavelengths from Figure 2a.

In this article, we combine two semi-classical approaches towards microscopic electron dynamics into a single feasible framework to address quantum corrections in MNPs allowing the description of isolated particles, clusters and large-scale (two- or three-dimensional) devices via the integration of analytical expressions into standard procedures. We hereby focus on results on damping in MNPs derived from the microscopic Random Phase Approximation (RPA), stemming from Lorentz friction, and spatial dispersion (nonlocal) effects obtained with the hydrodynamic approach. We discuss briefly the separate ingredients of these approaches in the next sections and give more details in the methods section. Moreover, we compare and combine the different processes of mesoscale electron dynamics stemming from scattering, Figure 1a,b, irradiation (Lorentz friction), Figure 1c, and nonlocal interaction, Figure 1d, and study their impact on the optical response of isolated MNPs and dimers. An emphasis is put on the size regimes where these effects are dominant for the materials silver, Figure 2, as well as for aluminum and gold, Figure 3.

## 2. Results

We briefly discuss classical electrodynamics and mesoscopic electron dynamics obtained from the RPA and GNOR theories. In summary, we compare quantum correction models stemming from microscopic RPA derivations with the following, semi-classical damping expressions

(1a)γ=γp,(Mie)(1b)γ=γp+CvFa,(Kreibig)(1c)γ=γp+CvFa+ω13ω1ac3,(perturbative)(1d)γ=Im(Ω2)=−13l+1+6lq2233lA+A2136l,(Lorentz),

and nonlocal interaction effects. Both approaches are described in more detail in the next sections and the methods section. The advantage in the analytic formulation is the straightforward integration with existing computational tools for nanospheres using modified Mie simulations and multiple scattering techniques [67] for clusters thereof or commercial software such as COMSOL (http://www.comsol.com).

### 2.1. Classical and Phenomenological Approaches

Typically, the optical response of a metal is described with the Drude model via the frequency-dependent permittivity

(2) $$\begin{array}{c} \epsilon \left(\omega \right)=\epsilon_{b}-\frac{\omega_{p}^{2}}{\omega (\omega +i\gamma_{p})}, \end{array}$$

where $\epsilon_{b}$ is the background permittivity given by bound (valence band) electrons, $\omega_{p}^{2}=4\pi n_{0}e^{2}\;/\;m$ is the plasmon frequency, determined by the material dependent electron density $n_{0}$ and mass *m*, and $\gamma_{p}$ is the inherent (bulk) damping rate. This widely used Drude model applies only to bulk material and should be modified for nanostructures to include effects due to the finite size of the system. One of the corrections considered by Kreibig and von Fragstein [68] is the inclusion of an additional damping due to the scattering on the physical particle boundaries, depicted in Figure 1b. This is in particular important in particles of sizes equal or smaller than the mean free path $\lambda_{b}$ of electrons in bulk metal. In such a case, the electrons will experience (in the classical picture) additional scattering from the boundary of the system. Mathematically, it is described as $\gamma_{K}=v_{F}\;/\;L_{eff}$ , where $v_{F}$ is the Fermi velocity of the electron gas and $L_{eff}$ is the effective mean free path of electrons resulting from collisions with the particle surface [38,68,69]. The common feature is that $L_{eff}$ reflects the volume (proportional to the number of electrons inside the nanoparticle) to surface ratio of the particle. According to this, we get the $\gamma_{K}\left(a\right)=Cv_{F}\;/\;a$ , where *a* is the radius of the nanoparticle and *C* is a constant of the order of unity which depends on the scattering type and particle radius. Similarly, collision effects in the bulk, depicted in Figure 1a, can be described via the damping term $\gamma_{p}v_{F}\;/\;2\lambda_{b}$ .

### 2.2. Random Phase Approximation

Nevertheless, this phenomenological approach neglects the microscopic dynamics of electrons inside the MNP. Their accelerated movement (plasmon oscillation) leads to energy loss via irradiation of the electromagnetic field, see Figure 1c. In case of nanoparticles much smaller than the incident wavelength, this effect can be expressed by the Lorentz friction, an effective field stemming from the plasmon induced dipole field D(t) as $E_{L}=2\;/\;3c^{3}\partial^{3}D\left(t\right)\;/\;\partial t^{3}$ , with *c* being the speed of light [70]. The dynamics of the electron density can be described using a driven, damped oscillator, with the incident electromagnetic wave being the driving force and the damping arising form electron scattering (bulk $\gamma_{p}$ and Kreibig damping $\gamma_{K}$ ) and electromagnetic field irradiation (Lorentz friction).

An analytical form of the exact solution for the damping γ and self-frequency $\omega_{L}$ (the exponents $\Omega_{i}$ of solution ∼ $e^{i\Omega_{i}t}$ for self-modes *i*) including Lorentz friction exists [61], which is discussed in more detail in the methods section. They can be summarized as follows

(3) Ω1=−i3l−i21/3(1+6lq)3lA−iA21/33l∈Im,Ω2=−i3l+i(1+i3)(1+6lq)22/33lA+i(1−i3)A21/36l=ωL+iγ,Ω3=−ωL+iγ=−Ω2*,

where $A={\left(B+\sqrt{4{\left(-1-6lq\right)}^{3}+B^{2}}\right)}^{1/3}$ , $B=2+27l^{2}+18lq$ , $q=\frac{1}{\tau_{0}\omega_{1}}$ , $l=\frac{2}{3\sqrt{\epsilon_{0}}}{\left(\frac{a\omega_{p}}{c\sqrt{3}}\right)}^{3}$ and $1/\tau_{0}=\gamma_{p}$ . Exact inclusion of the Lorentz friction indicates that the radiative losses and the self-frequencies are a complicated function of particle radius as given by Equation (3), see the methods section for a detailed discussion.

Direct comparison to experimental work for this framework is available within Refs. [61,62,63,64] and good agreement has been found.

### 2.3. Nonlocal Optical Response

Aside from electron irradiation due to Lorentz friction, we discuss spatial dispersion (nonlocality) which denominates the effects of electron coupling over a short distance, see Figure 1d [40]. Such interactions are inherent to the solution for the displacement field D of the Coulomb equation

(4) $$\begin{array}{c} \nabla D\left(\omega ,r\right)=0\Rightarrow \;D\left(\omega ,r\right)=\int dr^{\prime }\epsilon \left(\omega ,r,r^{\prime }\right)E\left(\omega ,r^{\prime }\right). \end{array}$$

In homogeneous media, we can assume a dependence on the distance $\left|r-r^{\prime }\right|$ rather than on the specific position of electrons, which allows solving Maxwell’s equations in Fourier space D(ω,k)=ϵ(ω,k)E(ω,k) .

The dependence on the wave vector k enables us to describe nonlocal electron-electron interaction (Coulombic force) and electron diffusion effects. It is important to note that the large-*k* response that originates in the subwavelength oscillations of plasmonic excitations is not only an inherent prerequisite for many intriguing wave phenomena, but also particularly sensitive to nonlocality. However, the common Mie result has no upper wavelength cut-off and does suppress short-range electron interactions which can strongly dampen the response beyond $\omega /v_{F}$ . We show in the corresponding section below that accounting for nonlocal response leads to longitudinal pressure waves as additional solutions to the combined system of differential equations of the electromagnetic wave equation and (linearized) Navier-Stokes equation. This is in contrast to the damping expressions derived by Kreibig and for Lorentz friction. Such additional waves offer further damping channels, however, they can also support resonant enhancement effects [12,51,59,71].

Experimental work focusing on the blueshift found for nanoparticles decreasing in size, as well as the influence of the electron-spill out has been studied in Refs. [22,23,24,25,26,27,28,29], including comparisons with the hydrodynamic model.

### 2.4. Remarks on Retardation, Multipolar Response and Computational Feasibility

Both of the presented semi-classical approaches towards microscopic corrections in the mesoscale electron dynamics in metal nanoparticles have the advantage of analytic expressions fully compatible with existing computational procedures. For the quantum confinement picture of Kreibig and the mesoscopic RPA result for the Lorentz friction, modified damping terms were derived, see Equations (1a)–(1d), which can be used to directly replace the damping in the Drude expression for the permittivity given in Equation (2) and subsequently be used in standard Mie calculations and procedures to calculate optical properties of complex structures, e.g., with a multiple scattering approach [67] or within commercial software such as COMSOL.

It is important to note that although all electrons participate in plasmon oscillations, part of their irradiation is absorbed by other electrons in the system. This is in analogy with the skin-effect [72] in metals and introduces an effective radiation active electron layer of the depth h∼1/σω ( σ is the conductivity) underneath the particle surface. Therefore, the effective energy transfer outside of the nanoparticle will be reduced by the factor $\frac{4\pi }{3}\left(a^{3}-{\left(a-h\right)}^{3}\right)\;/\;\frac{4\pi a^{3}}{3}$ . According to this, we expect a decrease of radiative damping, especially for larger particles.

The nonlocal theory introduces a novel type of electron motion, longitudinal pressure waves, in addition to the transversal modes stemming from the classical electromagnetic wave equation. This additional electronic excitation offers further damping channels due to the energy lost in dampened motion. Here, the Mie coefficients are derived from the coupled system of optical and electronic excitation yielding modified scattering matrices that can again be implemented in existing methods. The properties of the longitudinal wave are given by analytic expressions such as their wave vector and their importance with respect to the common Mie solution is entirely captured in a single additional term, see the methods section for details.

Retardation is important when either the particle radius or the overall system size becomes large, i.e., for particle dimers, clusters and arrays. Although the presented microscopic effects are highly localized, they can have a strong impact on a larger particle or system in the interplay with long-range retardation effects. In addition, particle layer modes can couple to nonlocal modes within particle arrays and thus increase their impact on a larger scale [59,71]. It is thus noteworthy that the hydrodynamic theory and the damping terms stemming from microscopic analysis within the RPA allow fully retarded calculations; equally for planar geometries (nonlocal Fresnel coefficients) [51] and regular, two-dimensional particle arrays [41,59,71] and even charge carriers in electrolytes (*Nonlocal Soft Plasmonics*) [12].

## 3. Discussion

### 3.1. Single Metal Nanoparticles

We compare the quantum correction models introduced in the previous section, see Figure 1, as well as the combined effect of Kreibig damping Equation (1b), Lorentz friction Equation (1d) and spatial dispersion to classical Mie calculations for the materials gold, aluminum and silver in Figure 2 and Figure 3. Hereby, we show the effect on the Localized Surface Plasmon Resonance (LSPR) for all materials in Figure 2a, confirming that the modified damping rates do not alter the resonance position predicted by the classical calculations, whereas nonlocal response—and in combination with any damping model—does predict an increasing blueshift of the nanoparticle resonance with decreasing particle size. Looking at the extinction cross section as a function of particle radius in Figure 2b for silver and Figure 3 for gold and aluminum, we find that all correction models result in a reduction of the optical response in dependence of both the material and particle size, typically yielding a different optimized particle size. Hereby, Kreibig damping with a ∼ 1/a dependence drastically attenuates the optical response for the smaller size regime below the maxima (15 nm for Ag, 20 nm for Au, and 10 nm for Al), while the complex size dependence of Lorentz friction results in a greater effect above this particle size. The diffusion coefficient in the hydrodynamic (GNOR) model (imaginary part of the nonlocal parameter $\beta_{\mathrm{GNOR}}$ ) is chosen thus that its dampening effect captures the Kreibig result [56]. This is best seen in Figure 3a for Au. The hydrodynamic pressure (real part of the nonlocal parameter $\beta_{\mathrm{GNOR}}$ ) describes Coulomb interaction between electrons and results in the blueshift observed in Figure 2a at very small particle sizes below 5 nm. We can further incorporate the analytical expressions for Lorentz friction. This combined result shows the strongest attenuation since all different damping channels are included. At a larger particle size (60 nm for Ag, 80 nm for Au, and 40 nm for Al) all material models converge with classical Mie theory where the mesoscale electron dynamics cease to have an impact.

The damping associated with the Lorentz friction can be approximated to the simpler perturbative expression Equation (1c) in a narrow size window, see the methods section for a detailed discussion. Since the exact solution can be obtained with analytical expressions which can be incorporated into standard calculation schemes, we discuss exclusively exact Lorentz friction results.

**Figure 4:**
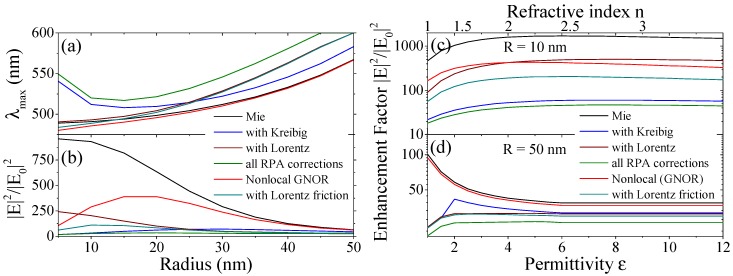
Maximum enhancement factor $\mathrm{EF}={\left|E\right|}^{2}\;/\;|E_{0}{|}^{2}$ at the particle surface for gold. Dependence of (**a**) the maximum EF and (**b**) its wavelength position for the different quantum corrections on the particle radius in water. (**c**), (**d**) The same as a function of the permittivity $\epsilon_{0}$ of the surrounding medium for nanospheres of (**c**) R=10 nm and (**d**) R=50 nm.

We study the (maximum) field enhancement factor $\mathrm{EF}={\left|E\right|}^{2}/{\left|E_{0}\right|}^{2}$ just outside of the NP ( r→a+ ) for the different damping models in Figure 4 for gold nanospheres. Hereby, Figure 4a shows the spectral position of the field maximum. The local field enhancement reveals the size dependence of the field resonance with the damping rates. It should be emphasized that Kreibig damping shows a strong redshift for small particle sizes of the spectral position of local field enhancement maxima in contrast to experimental findings [25,26,27] and approaches the Mie result for larger sizes. Nonlocal optical response agrees with the blueshift of the plasmon resonance found experimentally for noble metals, as already seen in the extinction cross section, Figure 2a. However, in order to correctly describe simple metals, the inclusion the electron spill-out region [52,57,65] is crucial. Furthermore, advances towards the spatial dispersion found in (doped) semiconductors were made recently [73,74], which is of further interest when using dielectric nanoparticles to enhance the performance of photovoltaic devices.

Lorentz friction is closest to the classical calculation for smaller sizes and deviates stronger at larger sizes. This is in agreement with the findings of Figure 2 and Figure 3. The corresponding field enhancement, shown in Figure 4b for gold MNPs in water, is strongly suppressed for the considered particle size range when including the damping models while spatial dispersion by itself reduces the predicted field enhancement mostly for smaller particle sizes and converges with the classical Mie result rapidly with increasing particle size. This behavior is corrected by incorporating Lorentz friction into the GNOR result.

Figure 4c,d shows the (maximum) field enhancement of gold nanoparticles in dependence of the refractive index (RI) of the surrounding medium (from air n=1 to Si n=3.4 ) for two particle sizes. This is accompanied with a linear (in case of the nonlocal theory approximately linear) shift in the resonance wavelength towards longer wavelengths (not shown). With increasing RI of the host medium, the enhancement factor reaches a saturation value which for increasing particle size converges for all material models discussed. The discrepancy between the local field enhancement values predicted remains similar for small MNPs in different host media spanning several orders of magnitude.

**Figure 5:**
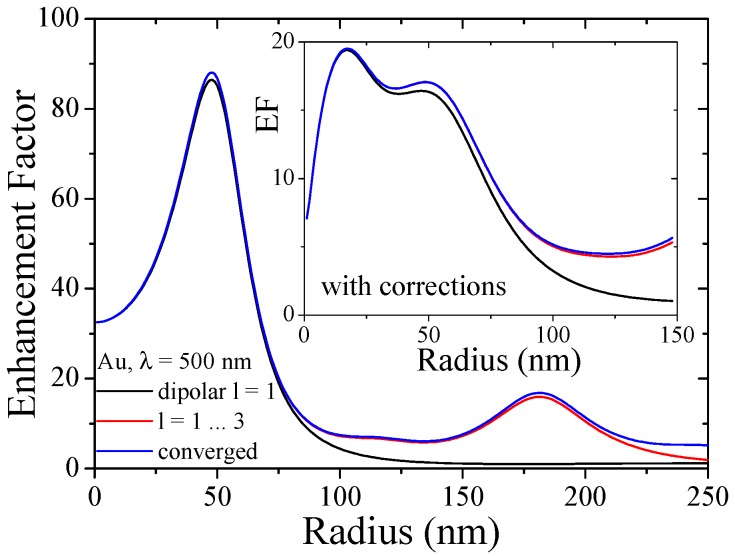
Size regime for multipolar response in metal nanoparticles. Enhancement factor $\mathrm{EF}={\left|E\right|}^{2}\;/\;|E_{0}{|}^{2}$ at the particle surface, where the EF is maximized, for λ=500 nm close to the corresponding Mie resonance of gold with classical Mie coefficients and as inset with combined microscopic corrections. The calculations are based on the dipolar response (black), the first three multipoles (red) and the converged result (blue).

The complexity of the Lorentz friction makes it necessary to restrict ourselves to the dipolar response of the plasmon oscillation. It is therefore important to consider the material, particle size and wavelength regime in order to assess whether the dipolar response model is adequate for the system under study. We show in Figure 5 for Au NPs the dipolar and the converged result of local field enhancement obtained from classical Mie calculations at a fixed frequency close to the respective plasmon resonance. Here, the dipolar approximation is valid up to ca. 100 nm in particle radius which in general covers the discussed microscopic effects well. The inset in Figure 5 compares this for the combined theories showing small differences already for particles above 25 nm radius.

### 3.2. Dimers

**Figure 6:**
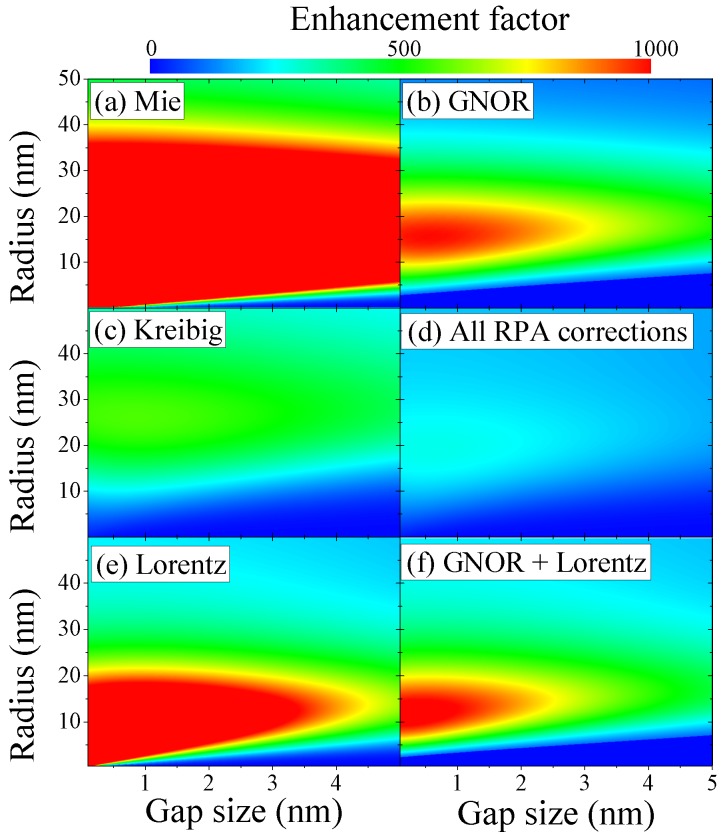
Impact of microscopic electron dynamics on gold dimers in water. We show the maximum field enhancement at the gap center of gold dimers dispersed in water in dependence of their radius ( a>0.5 nm) and separation (>0.1 nm) for (**a**) classical Mie calculations, (**b**) spatial dispersion with GNOR, (**c**) Kreibig damping, (**d**) all RPA corrections combined, (**e**) Lorentz friction and (**f**) GNOR with Lorentz friction. The incident field is polarized along the dimer axis and the maximum EF is evaluated at the respective resonance frequency calculated for each case.

For particle dimers, in addition to their size, the particle distance becomes important and retardation effects cannot be neglected for larger particles in close proximity. This can transfer the impact of localized microscopic electron dynamics onto a larger structure. Figure 6 shows the (maximum) field enhancement at the center of a gold dimer in water as a function of both particle size and distance for the different theories considered. The impact of nonlocal response, Figure 6b, on the classical Mie theory, Figure 6a, is visible as strong quenching of the local fields. It is worth remembering that one main effect is a blueshift in the position of the maximum enhancement factor, see again Figure 4a and Ref. [41]. In addition, the maximum field enhancement within the parametric area of particle and gap size is EF ≈9000 for the Mie calculations and EF ≈3000 for the nonlocal theory, showing that indeed there is an impact of the longitudinal waves found. The damping observed within Kreibig theory, Figure 6c, is dramatic for the dimer setup and the dominant contribution in the combined theory as seen in Figure 6d. This is also evidenced by comparing the Lorentz friction with and without nonlocal damping, see Figure 6e,f, respectively. The Lorentz friction has a strong impact on the optical response for larger particle sizes, but also dampens the dimer setup for increasing gap size, which points towards retardation and the increasing structural size as the main source for this damping effect. This leads to slightly stronger damping when combined with the additional plasmon quenching within GNOR in Figure 6f.

The strong field quenching poses limitations to the photovoltaic effect in solar cells. However, considering different materials for MNPs and their environment, the size regimes where local field quenching is dominant can be avoided with the presented theory of combined damping.

### 3.3. Summary

In conclusion, we have presented a number of semi-classical corrections to incorporate electron dynamics and non-classical interaction effects into optical response calculations for nanoparticles. Hereby, pure damping models, such as the Kreibig damping and Lorentz friction, derived from microscopic RPA theory, show an intriguing dependence on the particle size, where the material influences relevant size regimes. On the other hand, semi-classical nonlocal theories allow evoking additional modes in the system by explicitly considering mesoscopic dynamics of free electrons. This results in a correction of the spectral position of resonant phenomena and introduces additional, implicit damping channels. The phenomenological Kreibig damping does yield a plasmon broadening that agrees with experiments [38], however, it also introduces a redshift of the resonance with respect to the classical Mie result contrary to measurements on nanoparticles [25,26,27,29]. This is addressed by using the hydrodynamic GNOR (generalized nonlocal optical response) approach, i.e., by introducing a diffusion parameter, able to reproduce the Kreibig damping while fully capturing the observed plasmon broadening.

An important aspect is that the resulting analytical expressions can be implemented into existing computational procedures in a straightforward manner, as isolated theories or combined, allowing the comparison to experiments with little added numerical effort. We have studied the combined effect of these mesoscopic electron interaction effects for single nanospheres and gold dimers and have evidenced the importance of retardation as a way to communicate localized quantum effects and impact a larger structure.

The straightforward inclusion of electro-optical effects at the nanoscale into (metal) nanoparticle systems is of importance in nanostructures employed for photovoltaics and catalysis as well as in spectroscopy and sensing applications.

## 4. Methods

### 4.1. Electron Dynamics within the RPA

The model of electron dynamics inside MNPs [60,61,62] presented here is an extension to the RPA theory developed by Pines and Bohm [75] for bulk metals. In our model, a finite, rigid jellium defines the shape of a nanoparticle. The plasmon oscillations are described as local electron density fluctuations $\hat{\rho }\left(r,t\right)$ obtained from the Heisenberg equation

(5) $$\begin{array}{c} \frac{d^{2}\hat{\rho }\left(r,t\right)}{dt^{2}}=\frac{1}{{\left(i\hbar \right)}^{2}}\left[\left[\hat{\rho }\left(r,t\right),{\hat{H}}_{e}\right]{\hat{H}}_{e}\right] \end{array}$$

with a corresponding Hamiltonian ${\hat{H}}_{e}$ for electrons inside the MNP in the jellium model taking the following form

(6) $$\begin{array}{c} {\hat{H}}_{e}=\sum_{j=1}^{N_{e}}\left[-\frac{\hbar^{2}\nabla_{j}^{2}}{2m}-e^{2}\int \frac{n_{e}\left(r\right)d^{3}r}{|r_{j}-r|}\right]+\frac{1}{2}\sum_{j\neq j^{\prime }}\frac{e^{2}}{|r_{j}-r_{j^{\prime }}|}. \end{array}$$

The operator of the local electron density is defined as

(7) $$\begin{array}{c} \rho \left(r,t\right)=\langle \Psi_{e}\left(t\right)|\sum_{j}\delta \left(r-r_{j}\right)|\Psi_{e}\rangle \end{array}$$

where $\Psi_{e}$ is the electron wave function, $N_{e}$ is the number of collective electrons, $r_{j}$ and *m* are their positions and mass. The ion field is approximated as averaged background charge density and described as $n_{e}\left(r\right)|e|=n_{e}\Theta \left(a-r\right)\left|e\right|$ , where Θ is the Heaviside step function, *a* is the radius of the MNP and $n_{e}=N_{e}\;/\;V$ .

The first term in the Hamiltonian stands for the kinetic energy of electrons, the second for interaction between electrons and positive background charges (approximating the ion lattice potential) and the last for electron-electron Coulomb interaction.

Taking into account the sharp form of the positive charge density $n_{e}\left(r\right)$ , one can decompose Equation (5) into two parts corresponding to the inside and outside of the NP, which leads to two separate solutions describing the surface and bulk plasmons. This description is valid for NPs larger than ca. 5 nm for which the surface is well defined and the spill-out effect is negligible.

(8) $$\begin{array}{c} \delta \tilde{\rho }\left(r,t\right)=\left\{\begin{array}{c} \delta \tilde{\rho_{1}}\left(r,t\right),\;\mathrm{for}\;r<a,\\ \delta \tilde{\rho_{2}}\left(r,t\right),\;\mathrm{for}\;r\geq a,(r\to a+) \end{array}.\right. \end{array}$$

The electron density fluctuations are then described with the formulas

(9) $$\begin{array}{c} \frac{\partial^{2}\delta \tilde{\rho_{1}}\left(r,t\right)}{\partial t^{2}}=\frac{2}{3}\frac{\epsilon_{F}}{m}\nabla^{2}\delta \tilde{\rho_{1}}\left(r,t\right)-\omega_{p}^{2}\delta \tilde{\rho_{1}}\left(r,t\right) \end{array}$$

and

(10) $$\frac{\partial^{2}\delta \tilde{\rho_{2}}\left(r,t\right)}{\partial t^{2}}=-[\frac{2}{3}\frac{\epsilon_{F}}{m}\frac{r}{r}\nabla \delta \tilde{\rho_{2}}\left(r,t\right)+\;\frac{\omega_{p}^{2}}{4\pi }\frac{r}{r}\nabla \int d^{3}r_{1}\frac{1}{|r-r_{1}|}\left(\delta \tilde{\rho_{1}}\left(r_{1},t\right)\Theta \left(a-r_{1}\right)+\delta \tilde{\rho_{2}}\left(r_{1},t\right)\Theta \left(r_{1}-a\right)\right)]\delta \left(a+\varepsilon -r\right)\;\begin{array}{c} -\frac{2}{3m}\nabla \left\{\left[\frac{3}{5}\epsilon_{F}n_{e}+\epsilon_{F}\delta \tilde{\rho_{2}}\left(r,t\right)\right]\frac{r}{r}\delta \left(a+\varepsilon -r\right)\right\}. \end{array}$$

where $\epsilon_{F}$ is the Fermi energy.

The structure of the above equations is of an harmonic oscillator, which allows including a damping term in phenomenological manner by adding to the right hand side $-2\;/\;\tau_{0}\partial {\tilde{\rho }}_{1(2)}\left(r,t\right)\;/\;\partial t$ . The damping $2\;/\;\tau_{0}=\gamma_{p}+\gamma_{K}$ includes collision effects and Kreibig damping due to the particle boundary.

Assuming homogeneity of the external electric field E(t) inside the NP (dipole approximation), the solution for surface modes reduces to a single dipole mode

(11) $$\begin{array}{c} \delta \tilde{\rho }\left(r,t\right)=\sum_{m=-1}^{1}Q_{1m}Y_{1m}\left(\Omega \right),\;\mathrm{for}\;r\geq a,(r\to a+) \end{array}$$

and for bulk modes $\delta \tilde{\rho }\left(r,t\right)=0$ where r<a .

The function $Q_{1m}\left(t\right)\;\left(m=-1,0,1\right)$ represents dipole modes, $Y_{lm}\left(\Omega \right)$ is the spherical function. The former can be related to the vector q(t) via $Q_{11}=\sqrt{8\pi \;/\;3}q_{x}\left(t\right)$ , $Q_{10}=\sqrt{4\pi \;/\;3}q_{x}\left(t\right)$ , $Q_{1-1}=\sqrt{8\pi \;/\;3}q_{y}\left(t\right)$ satisfying the equation

(12) $$\begin{array}{c} \left[\frac{\partial^{2}}{\partial t^{2}}+\frac{2}{\tau_{0}}\frac{\partial }{\partial t}+\omega_{1}^{2}\right]q\left(t\right)=\frac{en_{e}}{m}E\left(t\right). \end{array}$$

Then the plasmon dipole can be defined as

(13) $$\begin{array}{c} D\left(t\right)=e\int d^{3}rr\delta \rho \left(r,t\right)=\frac{4\pi }{3}eq\left(t\right)a^{3}. \end{array}$$

Knowing this, the damping caused by electric field irradiation can be simply added to the right hand side of Equation (12) as additional field $E_{L}=2\;/\;3c^{3}\partial^{3}D\left(t\right)\;/\;\partial t^{3}$ hampering charge oscillations and can be rewritten in the form

(14) $$\begin{array}{c} \left[\frac{\partial^{2}}{\partial t^{2}}+\omega_{1}^{2}\right]D\left(t\right)=\frac{\partial }{\partial t}\left[-\frac{2}{\tau_{0}}D\left(t\right)+\frac{2}{3\omega_{1}\sqrt{\epsilon_{0}}}{\left(\frac{\omega_{p}a}{c\sqrt{3}}\right)}^{3}\frac{\partial^{2}}{\partial t^{2}}D\left(t\right)\right]. \end{array}$$

**Figure 7:**
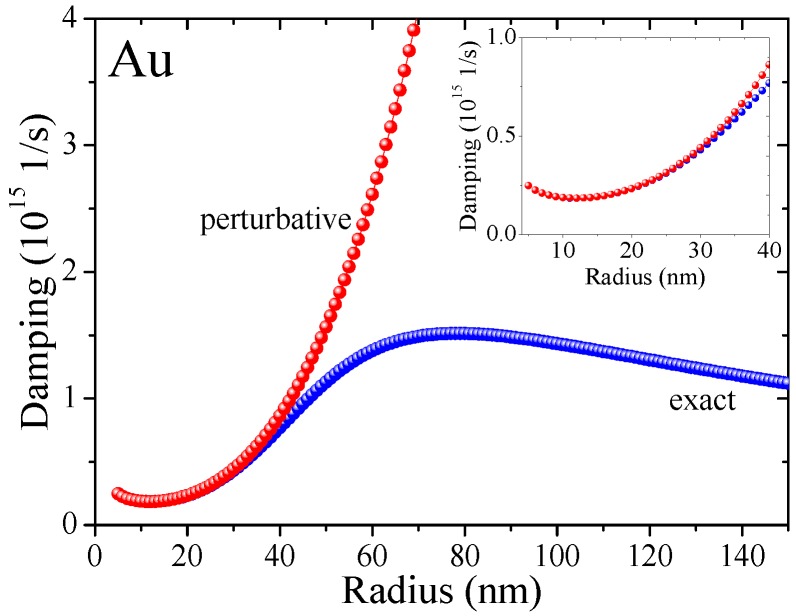
**Comparison of RPA damping rates.** The perturbative solution (red) and exact Lorentz friction (blue) for a Au nanoparticle in water.

The above equation is a third order linear differential equation and the exponents ∼ $e^{i\Omega_{i}t}$ of its solutions are given in Equation (3). A perturbation approach can be applied to Equation (14) for small particles using $\partial^{2}D\left(t\right)\;/\;\partial t^{2}=-\omega_{1}^{2}D\left(t\right)$ . Then the resulting damping term takes the form $\gamma =2\;/\;\tau_{0}+\left(\omega_{1}\;/\;3\sqrt{\epsilon_{0}}\right){\left(\omega_{a}\;/\;c\sqrt{3}\right)}^{3}$ . The comparison of both damping terms is shown in Figure 7 justifying the usage of the perturbation formulation for (gold) particles with radii up to ca. 30 nm, where the second term proportional to ∼ $a^{3}$ still fulfills the perturbation constrain.

For larger radii, the discrepancy between both solutions grows rapidly since the irradiation losses within the perturbation approach scale as $a^{3}$ . Therefore, the radiative losses dominate plasmon damping for large nanospheres. On the other hand, scattering is more important for smaller nanospheres scaling as $\frac{1}{a}$ . One can observe thus the size-dependent crossover in Figure 3a of the damping at ca. 12 nm for gold.

### 4.2. Electron Dynamics with the Hydrodynamic Model

In recent years, a great effort to theoretically [41,43,44,46,47,48,49,50,51,53,54,55,56,59] describe and subsequently to experimentally [25,26,27,29] verify the effect of spatial dispersion in metals was made. In the hydrodynamic approach, coupling the electromagnetic wave equation

(15) $$\begin{array}{c} \nabla \times \nabla \times E-k^{2}\epsilon_{b}E=\frac{4\pi ik^{2}}{\omega }j^{\mathrm{ind}} \end{array}$$

to the (linearized) Navier-Stokes equation

(16) $$\begin{array}{c} j^{\mathrm{ind}}=\frac{i}{\omega +i\gamma_{p}}\left(\frac{\omega_{p}^{2}}{4\pi }E-\left(\beta^{2}+D\left(\gamma_{p}-i\omega \right)\right)\nabla \rho^{\mathrm{ind}}\right) \end{array}$$

allows treating the conduction band electrons as a plasma subject to short-ranged interaction such as the Coulomb force included in the pressure term $p=\beta^{2}\rho^{\mathrm{ind}}$ and electron diffusion via the diffusion coefficient *D*. It is convenient to abbreviate $\beta_{\mathrm{GNOR}}^{2}=\beta^{2}+D\left(\gamma_{p}-i\omega \right)$ (where GNOR refers to the Generalized Nonlocal Optical Response model [55,56]). With this, we can write the wave equation in a compact form

(17) $$\begin{array}{c} \nabla^{2}E+k^{2}\epsilon_{⊥}E=\eta \rho^{\mathrm{ind}}, \end{array}$$

where $\eta =4\pi \left(\frac{1}{\epsilon_{b}}-\frac{k^{2}\beta_{\mathrm{GNOR}}^{2}}{\omega (\omega +i\gamma )}\right)$ and $\epsilon_{⊥}=\epsilon_{b}-\omega_{p}^{2}\;/\;\omega (\omega +i\gamma_{p})$ . Together with the continuity equation $\nabla j^{\mathrm{ind}}=i\omega \rho^{\mathrm{ind}}$ , we readily obtain a separate wave equation for the induced charges

(18) $$\begin{array}{c} -\beta_{\mathrm{GNOR}}^{2}\nabla^{2}\rho^{\mathrm{ind}}=\frac{\epsilon_{⊥}}{\epsilon_{b}}\omega \left(\omega +i\gamma_{p}\right)\rho^{\mathrm{ind}}, \end{array}$$

where $\nabla E=4\pi \;/\;\epsilon_{b}\rho^{\mathrm{ind}}$ was used. This yields the wave vector of the longitudinal field and motion of electrons

(19) $$\begin{array}{c} q=\frac{1}{\beta_{\mathrm{GNOR}}}\sqrt{\frac{\epsilon_{⊥}}{\epsilon_{b}}\omega \left(\omega +i\gamma_{p}\right)}. \end{array}$$

Nonlocal theories predict finite distributions of induced charges at an illuminated metal surface—in contrast to classical electrodynamics—with a characteristic penetration depth Im(1/q) comparable to the electron spill-out [41,76].

Thus, this system of coupled equations yields an additional wave solution, longitudinal in character, and can be solved for different geometries leading to nonlocal extensions of Mie [41,48] and Fresnel coefficients [51], including for charge carriers in electrolytes [12]. Typically, hard-wall boundary conditions are assumed for the additional boundary condition $\hat{n}j^{\mathrm{ind}}\equiv 0$ prohibiting electrons to trespass through the particle surface into the dielectric surrounding, using a uniform electron density $n_{0}=\omega_{p}^{2}m\;/\;4\pi e^{2}$ inside the material and neglecting the electron spill-out. However, it was shown that a smooth surface distribution of electrons can be taken into account accurately [57,65] and that the hydrodynamic model is capable of dealing with the spill-out by solving the above equations with position-dependent material parameters $\omega_{p}{\left(z\right)}^{2}=4\pi n_{0}\left(z\right)e^{2}\;/\;m$ .

The main observations of nonlocal theories are a blueshift of the plasmon resonance with respect to the common local approximation and plasmon broadening, in particular tied to the diffusion coefficient which can be set to fully capture the broadening found with Kreibig damping [55,56]. In the present work, we have adopted the diffusion coefficients as deduced in Ref. [56] for the different materials, reflected, for instance, in the correspondence between the Kreibig and GNOR result for gold in Figure 3a. Moreover, we add the Lorentz friction result from the RPA technique summarized in Equation (1d) to our GNOR calculations.

Next, we present the derivation of nonlocal Mie scattering coefficients of individual spheres and nanoshells described with the hydrodynamic model [41] starting from Equation (17) which describes the evolution of the electric field, together with Equation (18) which is the wave equation for the induced charge. The resulting scattering matrices can be used to investigate interacting spheres with a multiple scattering method [67]. The hydrodynamic model has no free parameters which makes the resultant nonlocal response for the short distances involved in the interaction (Coulomb force, diffusion) between the charges of MNPs the sole source of these effects, in contrast to the quantum-confinement picture for plasmon broadening presented by Kreibig.

It is convenient to use an expansion of the electric field into scalar functions [77] as

(20) $$\begin{array}{c} E=\left(1/k\right)\nabla \psi^{L}+L\psi^{M}+\frac{\nabla \times L}{ki}\psi^{E}, \end{array}$$

where L=−ir×∇ is the angular momentum operator, and the superscripts *E*, *M*, and *L* indicate electric, magnetic, and longitudinal components, respectively. The additional boundary condition, Equation (16), becomes with $\hat{r}j=0$

(21) $$\begin{array}{c} \beta_{\mathrm{GNOR}}^{2}\frac{\partial }{\partial r}\rho^{\mathrm{ind}}=\frac{e^{2}n_{0}}{mk}\left(\frac{\partial }{\partial r}\psi^{L}+\frac{1}{r}l\left(l+1\right)\psi^{E}\right) \end{array}$$

in terms of the scalar functions and the angular momentum number *l* using the identity $-r\cdot \left(\nabla \times L\right)=\left(-ir\times \nabla \right)\cdot L=L^{2}=l\left(l+1\right)$ . The boundary conditions for the electric and magnetic field components result in the continuity of $\psi^{M}$ , $\left(1+r\frac{\partial }{\partial r}\right)\psi^{M}$ , $\psi^{L}+\left(1+r\frac{\partial }{\partial r}\right)\psi^{E}$ , and $\epsilon \psi^{E}$ for the scalar functions.

The magnetic and electric scalar functions $\psi^{\nu }\;\left(\nu =\left\{E,M\right\}\right)$ obey a Helmholtz equation of the form $\left(\nabla^{2}+k^{2}\epsilon_{⊥}\right)\psi^{\nu }=0$ and can therefore be expanded in terms of spherical Bessel functions $\psi^{\nu }=\sum_{L}\psi_{L}^{\nu }j_{L}\left(k_{⊥}r\right)$ . Similarly, the electron density is expanded into $\rho^{\mathrm{ind}}\left(r,\omega \right)=\sum_{L}\rho_{L}j_{L}\left(qr\right)$ , with the longitudinal wave vector *q* given by Equation (19). The longitudinal scalar function satisfies a different wave equation, namely $\nabla^{2}\psi^{L}=4\pi k/\epsilon_{b}$ , which we find from the Coulomb law $\nabla \epsilon_{b}E=4\pi \rho^{\mathrm{ind}}$ .

Note that the above analysis is needed for the metal region, where the electric ( ν=E ) and magnetic ( ν=M ) field are given by $A_{l}^{\nu }j_{L}$ , with $j_{L}=j_{lm}\left(k_{⊥}r\right)$ . Outside the particle, the longitudinal scalar function vanishes since there are no induced charges in the dielectric surrounding. Therefore, the electric scalar field is given by $j_{lm}\left(k_{0}r\right)+t_{l}^{\nu }h_{lm}^{+}\left(k_{0}r\right)$ with unknown parameters $A_{l}^{\nu }$ and scattering matrix $t_{l}^{\nu }$ . Exploiting the boundary conditions stated above, we find a set of linear equations for the magnetic and electric scattering matrices. Interestingly, the magnetic scattering matrix is unchanged with respect to the local theory, indicating that magnetic modes are not sensitive to the induced longitudinal modes. The scattering matrix for the electric scalar function is more complicated than in the local approximation due to the appearance of $\psi^{L}$ in the metal region that contains information on the nonlocal response. The additional boundary condition yields a prescription to calculate $\rho_{L}$ .

The local scattering matrix can then be extended by a single parameter describing nonlocal behavior of the electron motion in the conduction band

(22) $$\begin{array}{c} g_{l}=\frac{l\left(l+1\right)j_{l}\left(\theta_{⊥}\right)j_{l}\left(qa\right)}{qaj_{l}^{\prime }\left(qa\right)}\left(\frac{\epsilon_{⊥}}{\epsilon_{b}}-1\right) \end{array}$$

and becomes with $\theta_{0}=ka\sqrt{\epsilon_{0}}$ and $\theta_{⊥}=ka\sqrt{\epsilon_{⊥}}$ .

(23) $$\begin{array}{c} t_{l}^{E}=\frac{-\epsilon_{⊥}j_{l}\left(\theta_{⊥}\right){\left[\theta_{0}j_{l}\left(\theta_{0}\right)\right]}^{\prime }+\epsilon_{0}j_{l}\left(\theta_{0}\right)\left({\left[\theta_{⊥}j_{l}\left(\theta_{⊥}\right)\right]}^{\prime }+g_{l}\right)}{\epsilon_{⊥}j_{l}\left(\theta_{⊥}\right){\left[\theta_{0}h_{l}^{+}\left(\theta_{0}\right)\right]}^{\prime }-\epsilon_{0}h_{l}^{+}\left(\theta_{0}\right)\left({\left[\theta_{⊥}j_{l}\left(\theta_{⊥}\right)\right]}^{\prime }+g_{l}\right)}, \end{array}$$

where the primes indicate differentiation with respect to the θ variables. The scattering coefficients $t_{l}^{\nu }$ fully contain the optical response of the particle for an external observer.

Note that the nonlocal parameter *g* vanishes under the assumption of local response ( $\beta_{\mathrm{GNOR}}\to 0\Rightarrow g_{l}\to 0$ ) fully recovering the original Mie coefficients [15,78]. This allows us to study the electro-optical properties of NPs with only a small correction in available numerical procedures, see for instance Figure 5.

Likewise, for a nonlocal metal nanoshell, the magnetic response is insensitive to the nonlocal properties of the material. The electric part, however, mixes with the longitudinal components from the two interfaces of the metal intermediate layer. For the electric scalar functions, we obtain a linear system of six equations and analytical solutions exist for the metal nanoshell [41,79].

### 4.3. Simulations

The modeling presented in this article was obtained by both using the commercial software COMSOL Multiphysics (http://www.comsol.com) and in-house numerical code to evaluate Mie coefficients, from Equation (23).

To make predictions that can be compared to experiments, the expressions obtained are used to calculate e.g., the extinction cross section of an individual sphere via

(24) σext=2πk2ϵ0∑l(2l+1)Im(tlE+tlM).

Note that only the electric scattering matrix is sensitive to nonlocal contributions.

The scalar electric field is obtained from $j_{lm}\left(k_{0}r\right)+t_{l}^{E}h_{lm}^{+}\left(k_{0}r\right)$ outside the particle, with the corresponding spherical Bessel and Hankel functions and the related vector field from Equation (20).

The analytic damping expressions Equations (1a)–(1d) are directly introduced as damping terms in the permittivity of the different material permittivities, Equation (2).

For dimers, we use a multiple elastic scattering approach [67].
